# Polymorphisms of the oxytocin receptor gene and overeating: the intermediary role of endophenotypic risk factors

**DOI:** 10.1038/nutd.2017.24

**Published:** 2017-05-22

**Authors:** C Davis, K Patte, C Zai, J L Kennedy

**Affiliations:** 1 Kinesiology and Health Sciences, York University, Toronto, ON, Canada; 2 Department of Psychiatry, University of Toronto, Toronto, ON, Canada; 3 School of Public Health and Health Systems, University of Waterloo, Waterloo, ON, Canada; 4 Neurogenetics Department, Centre for Addiction and Mental Health, Toronto, ON, Canada

## Abstract

**Background/Objectives:**

Oxytocin (OXT) is an evolutionarily ancient neuropeptide with strong links to affiliative and prosocial behaviors, and the management of stress. Increases in OXT also tend to decrease food intake, especially of sweet carbohydrates. The social correlates of low OXT levels mesh with the social deficits and stress proneness identified in interpersonal models of overeating, as well as the increased appetite for highly palatable foods typically seen in chronic overeaters. The objectives of this study were to investigate links between polymorphisms of the oxytocin receptor (*OXTR*) gene and overeating, and to examine *OXTR* links with relevant endophenotypes of overeating related to reward and stress sensitivity, and to food preferences.

**Subject/Methods:**

The sample comprised 460 adults between the ages of 25 and 50 years recruited from the community, and representing a broad range of body weights. Overeating, reward and punishment sensitivity, and food preferences, were quantified as composite variables using well-validated questionnaires. In addition, seven single-nucleotide polymorphisms (rs237878, rs237885, rs2268493, rs2268494, rs2254298, rs53576, rs2268498) of the *OXTR* gene were genotyped.

**Results:**

Analyses identified a four-marker haplotype that was significantly related to food preferences. Individual genotype analyses also found that at least one of the markers was related to each of the phenotypic variables. In addition, an empirically derived structural equation model linking genetic and phenotype variables produced a good fit to the data.

**Conclusions:**

The results of this preliminary study have demonstrated that *OXTR* variation is associated with overeating, and with endophenotypic traits such as sweet and fatty food preferences, and reward and punishment sensitivity. In general, the genetic findings also favor the view that overeating may be associated with relatively low basal OXT levels.

## Introduction

Oxytocin (OXT) is an evolutionarily ancient neuropeptide, which influences a broad range of affiliative, and related survival behaviors.^1^ As such, OXT is popularly called the ‘love hormone’^2^ because it aids in the regulation of our sensitivity to reward, including parental attachment, pair bonding and empathy; and it facilitates use of these behaviors to reduce stress.^3, 4, 5^ In essence, increases in OXT tend to amplify one’s responsiveness to the importance of social cues so that both rewarding and aversive events become more impactful on the individual.^6^ Not surprisingly, the relationship between OXT and social behaviors is moderated by salient environmental factors.^2^ For example, OXT’s effects on prosocial behavior will depend on whether the context is affiliation-supportive and whether there are any social barriers to closeness.^2^

### OXT and food intake

In recent years, there has been a flurry of interest in the anorexigenic properties of OXT.^7^ For instance, it is well established that exogenous administration of OXT tends to decrease meal duration and to reduce food intake, especially of sweet carbohydrates.^7, 8^ It also appears that OXT selectively inhibits hedonic eating—rather than deprivation-induced eating—by diminishing brain-reward dopaminergic signaling.^9^ Moreover, there seems to be a specific functional relationship between OXT and sugar since an OXT antagonist stimulated sucrose intake at a 10-times lower dose compared with its effect on other carbohydrates such as glucose or on an artificial sweetener (saccharin).^10^ However, recent evidence suggests that OXT is really a ‘conditional anorexigen’, given that feeding in a social context has been shown to abolish its inhibitory effects on food intake.^11^

Endogenous OXT levels are also relevant to food intake and weight gain in a similar manner. In accord with its role in sugar-specific satiety, mice deficient in OXT also tend to overconsume sucrose solutions, both initially and over a sustained period of time.^12^ Similarly, it has been shown that *OXTR*-deficient mice tended to develop late-onset obesity.^13^ In addition, serum OXT levels were lower in obese adults and those with type 2 diabetes than in normal-weight controls.^14^ Relatedly, results from a post-mortem study of patients with Prader–Willi Syndrome (a disorder characterized by excessive food consumption and weight gain) showed a large reduction of OXT-expressing neurons in the paraventricular nucleus and smaller OXT cell volumes compared with their control counterparts.^15^

To date, studies of OXT signaling in the risk for, and development of, eating disorders are relatively scarce and mostly based on small-sample research.^16^ Of relevance, however, are the findings that chronic sucrose overconsumption tends to downregulate the anorexigenic OXT system in such a way that an elevated food-load ‘threshold’ is required to induce satiety.^17^ For instance, a recent study found that binge-prone rats consumed ~30% more sucrose in a non-stressful situation, and 60% more sucrose in a stressful environment, compared with their non-binge-prone counterparts—findings that may also be moderated by OXT levels.^18^ Likewise, there is good clinical evidence that chronic overeaters have higher reward responsiveness to palatable food, and tend to experience greater food cravings, compared with their normal-weight counterparts.^19^ Of relevance is the evidence that OXT is a well-established anxiolytic agent that tends to buffer cortisol responses to stress, especially in those with poor emotion-regulation abilities.^20^

### Interpersonal theories of overeating

It is striking that the social deficits associated with relatively low levels of OXT—such as attachment avoidance, relationship difficulties, parental insensitivity, and stress proneness—show remarkable similarities to the social impairments identified in interpersonal models of overeating.^21^ For instance, several studies have identified poor interpersonal skills, avoidance of emotional expression, interpersonal distrust and a diminished ability to cope with negative feelings across diverse samples of adults with clinically significant overeating.^22, 23^ Furthermore, significant links have been established between attachment avoidance and binge eating,^24^ and between attachment insecurity and increased risk for disordered eating in general.^25^ Binge eating in both mothers and fathers has also been associated with poor parent–child interactions during feeding and with the child’s emotional–behavioral problems over time.^26^ Similarly, overeating—especially among those who binge eat—has been associated with an elevated proneness to stress, and with poor emotional regulation.^27^ Interestingly, a large population study recently found that across all forms of disordered eating, patients were more likely to be childless than were their healthy counterparts with an odds ratio of 1.86.^28^ Such outcomes might have as much to do with relationship and social-bonding deficits as with poor physical reproductive health.

### An OXT-related model of overeating

Based on the body of research reviewed above, it is proposed that OXT may have important implications in the risk for, and development of, clinically significant overeating. Relatively low trait levels of OXT may foster a propensity to overconsume, especially in an environment where highly palatable temptations are ubiquitous, easily available and reasonably inexpensive, and among those with elevated sensitivity to the rewarding properties of such foods. Over time, chronic consumption of a sugar-laden diet may exacerbate the consumption of sweet substances. Relatively low OXT levels are also associated with a myriad of interpersonal deficits, which may contribute to elevated stress, particularly in those who are sensitive to social rejection and anxiousness. Taken together, these factors may foster increases in palatable food consumption as a means of emotional comfort. Such a stress-diathesis model of overeating meshes with the interconnected influences of the OXT system on complex human behaviors and personality traits.

**Figure 1:**
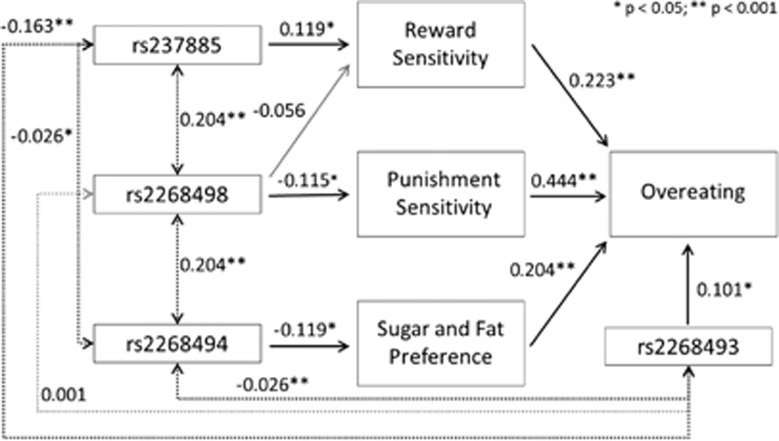
SEM (with standardized regression weights) showing relationships among *OXTR* polymorphisms, overeating and endophenotypic risk factors for overeating.

The aim of the current *exploratory* study was to examine links between markers of the *OXTR* gene and overeating. We also investigated associations between the *OXTR* markers and established endophenotypes of overeating such as sensitivity to both punishment and reward, and palatable food preferences.^29, 30^ In a relatively large sample of adult men and women, structural equation modeling (SEM) was used to assess simultaneously the relationships proposed in Figure 1.

## Materials and methods

### Participants and procedures

The sample consisted of 460 adults (females=346) between 24 and 50 years of age. Inclusion criteria were residence in North America for at least 5 years before enrollment in the study, and fluency in written and spoken English. Women were also required to be premenopausal as indicated by the self-report of a regular menstrual cycle. Exclusion criteria included serious medical illnesses such as cancer or diabetes, or severe physical disabilities such as cerebral palsy. Those with a current axis I diagnosed disorder, with the exception of unipolar depression, were also excluded. In addition, women with a pregnancy in the previous 6 months, or who were lactating, were not included. The majority of the sample was Caucasian (79%), with 15% identifying as African descent and the remainder as ‘Other’. The sample represented a broad range of body mass index (BMI) values (17.8–75.2 kg m^−2^).

Participants comprised a community-based sample and were recruited from posters, newspaper advertisements and online sites such as Craigslist and Kijiji in a large Canadian city. After a brief telephone screening, an appointment was made for each participant to visit the authors’ laboratory at a large clinical and research facility. Each participant was given the questionnaire package to complete. Height and weight were measured with the participant standing in stocking feet and wearing light indoor clothing, and a blood sample was taken at the on-site medical laboratory. The procedures used in this study were approved by the institutional Research Ethics Boards and were carried out in accordance with the Declaration of Helsinki.

### Selection of *OXTR* gene markers

Seven single-nucleotide polymorphisms (SNPs) in the *OXTR* gene, localized on 3p25.3, were selected for inclusion in this study based on their reported associations with the salience of, and sensitivity to, both rewarding and aversive social stimuli, and with other aspects of human social behavior.^31^ We also included SNPs with a demonstrated link to eating-related behaviors.^32, 33^

For instance, the G allele at the intron-3 of the rs53576 marker has been associated with higher reward dependence, increased amygdalar activation during the facial expression face-matching task,^34^ higher levels of trust and empathy,^35^ more sensitive parenting^36^ and increased sensitivity to social support.^37^ Additionally, the GG genotype carriers were more likely to report dieting to lose weight and less likely to eat sugary and fatty foods, such as cakes and puddings.^38^

At the intron-3 of the rs2268493 marker, the T allele has been associated with social impairment, autism spectrum disorders, and attention deficit/hyperactivity disorder.^39, 40, 41^ There is other evidence that this SNP may impact brain-reward circuitry. For instance, the TT genotype group displayed decreased activation in mesolimbic regions (including the nucleus accumbens and amygdala) in response to a monetary-rewards task.^3^ The T allele was also associated with poorer performance on a composite social cognition index.^42^

The A-allele carriers at another intron-3 marker, rs2268494, has been associated with reduced anger responses in a laboratory game focused on interpersonal interactions.^43^ Using a cumulative risk index including the AA genotype of this SNP, another recent study found less empathic concern to their romantic partner’s distress and lower social reciprocity in support-giving interactions.^44^

The G allele at the intron-3 of the rs2254298 marker has been associated with reduced social anxiety, decreased responsivity to stressors and lower social impairments.^45, 46^ Those carrying the G allele also showed lower amygdala response in tasks involving socially relevant face stimuli.^47^ In addition, a case–control study of patients with eating disorders found that A-allele carriers had a greater preoccupation with food and weight, and heightened social anxiety.^32^

The TT genotype at the intron-3 of the rs237885 marker has been broadly overrepresented in schizophrenia patients,^48^ and in another study, the TT genotype carriers displayed increased callous-unemotional traits compared with their counterparts.^49^ Of relevance, the prepulse inhibition of the startle reflex was significantly lower in the TT genotype.^50^ As low prepulse inhibition is associated with depression, these findings mesh with the results of the studies described above.

The T allele of the promoter rs2268498 marker has been associated with higher scores on a measure of empathy,^51^ and on a measure of social-perception abilities including the strength of social bonding, such as kinship and intimacy.^52^ The TT genotype was also associated with the lowest scores on measures of fear and sadness, as well as on negative emotionality.^53^

Finally, the C allele at the 3′ rs237878 marker has been associated with higher levels of extraversion—a personality construct with strong positive links to reward sensitivity (RS) and sensation seeking—compared with the T allele.^54^

### Questionnaire measures

The three psychological independent variables (reward sensitivity, punishment sensitivity and food-reward preferences), and the dependent variable (overeating), were operationally defined as separate composite variables (using principal components analysis) to reflect the multidimensionality of each construct.

#### Reward sensitivity

RS comprises three questionnaires, which were designed to assess *approach motivation*—one of three evolutionarily adaptive brain systems underlying current psychobiological theories of personality.^55^ This neurocircuitry regulates the incentive to obtain rewards as well as the hedonic experience of pleasure derived from these rewards.^56^ The 30-item Barratt Impulsivity Scale identifies facets of impulsiveness such as non-planning, and the tendency to act rashly and to make quick decisions.^57^ The Reward Subscale of the Sensitivity to Punishment and Sensitivity to Reward Questionnaire contains 24 forced-choice items assessing the respondent’s approach responses under various conditions of reward—including tangible rewards such as money and pleasure-inducing substances, as well social rewards such as receiving admiration from others.^58^ And finally, the 13-item Behavioral Activation Scale comprises three subscales, which collectively assess (i) anticipation of, and positive response to, rewards, (ii) the degree of persistence in achieving rewarding goals, and (iii) the strength of one’s desire for these rewards.^59^

#### Punishment sensitivity

PS includes three questionnaires, which reflect *avoidance motivation*—another evolutionarily adaptive brain system regulating the drive to avoid or retreat from situations of threat or punishment, and from other aversive events.^60^ The 35-item Harm Avoidance Scale of the Temperament and Character Inventory reflects the tendency to inhibit behavior in response to aversive or punishing stimuli.^61^ The Punishment Subscale (RS) of the Sensitivity to Punishment and Sensitivity to Reward Questionnaire contains 24 forced-choice items assessing the respondent’s avoidance responses in general situations involving worry and the threat of mistreatment, failure and the anticipation of non-reward.^58^ And finally, the 7-item Behavioral Inhibition Scale assesses both fear and anxiety responses to aversive social and environmental stimuli and situations.^59^

#### Food-reward preferences

Food-reward preferences were assessed by subscales of the well-validated Food Preference Questionnaire.^62^ This questionnaire provides hedonic ratings for 72 common food items arranged according to a 2 (FAT: high vs low) x 3 (OTHER MACRONUTRIENT: high simple sugar, high complex carbohydrate, and high protein) matrix with 12 items in each cell. In the current study, High Sugar and High Fat Foods is reflected by the 12 items from the high fat/high simple sugar cell; High Sugar Foods includes the 12 items from the High Simple Sugar/Low Fat cell and the High Fat Foods is the total (24 items) of the High Fat/High Carbohydrate and High Fat/High Protein subscales. The 72 food items of the Food Preference Questionnaire vary systematically and significantly with respect to their macronutrient content.

#### Overeating

Overeating comprises three scales representing different aspects of overconsumption of (mostly) highly palatable foods. *Binge Eating* was assessed by the five-item subscale of the Binge Eating Questionnaire, which includes frequency and severity of symptoms such as loss-of-control overeating, and negative affect following a binge.^63^ The Emotional Eating and Snacking on Sweets subscales (10 and 6 items, respectively) of the Eating Behavior Patterns Questionnaire were also included.^64^ The former assesses eating in response to negative moods and often in the absence of hunger. It also reflects the use of palatable food to comfort a distressed state. The latter describes an eating pattern characterized by the frequent consumption of sugary snacks throughout the day, and sometimes as a replacement for regular meals.

### Genotyping methods

Genomic DNA was purified from blood lymphocytes using the high-salt method as described previously.^65^ Genotyping was conducted on OpenArray plates (Life Technologies, Applied Biosystems Inc., Foster City, CA, USA) using a PCR-based method in the QuantStudio 12 K Flex Real-Time PCR System. We randomly regenotyped 10% of the samples for quality control purposes.

For the genetic analyses, departure from Hardy–Weinberg equilibrium was tested for each marker, and linkage disequilibrium (LD) values between markers were determined in Haploview version 4.2 (Broad Institute of MIT and Harvard, Cambridge, MA, USA).^66^ Single-marker analyses were conducted using SPSS (IBM Analytics), whereas haplotype analyses were performed with minimum haplotype frequency threshold of 0.05 in UNPHASED version 3.1.7.^67^

### Statistical analyses

The statistical analyses were conducted in two stages. Initially, four one-way analysis of variance procedures were conducted for each SNP, with genotype as the independent variable and RS, PS, food preferences, and overeating as separate dependent variables. These preliminary analyses identified relevant SNPs to include in the SEM illustrated in Figure 1. SEM procedures enable multiple regression equations to be tested simultaneously, which allows for a comprehensive model of the relationships between *OXTR* markers and endophenotypes associated with overeating. All parameters were determined *a priori* to testing. A model was tested in which RS, PS, and food preference each predicted overeating, with the SNPs added as predictors of the appropriate variables according to the significant effects identified in the preliminary analyses. The four factor scores were used instead of latent variables to avoid issues of collinearity in testing the SEM model, and covariances were added among the four *OXTR* SNPs given they were all markers on the same gene.

SEM was performed using SPSS AMOS 24. Missing data were handled using the AMOS estimate means and intercepts function, which applies maximum-likelihood methods.^68^ The *χ*^2^ test and a number of ‘approximate’ or ‘global’ fit tests were calculated. The *χ*^2^ analysis is known as the ‘exact-fit’ test in SEM, because a nonsignificant value requires the model-implied population covariance matrix to have zero discrepancies from the actual sample observed covariance matrix.

## Results

### Composite variables

Principal component analysis (SPSS version 23) was used to create the four composite variables, as described in the Methods section. Total scores, not individual items, were entered into the principal component analyses. Each analysis extracted only one component with an eigenvalue>1. Table 1 presents the component matrix for each composite variable showing factor loadings, and the percentage of variance accounted for by the extracted component. In all subsequent analyses, the derived factor scores were used to reflect the composite variables.

### Descriptive statistics

Prior to the analyses, all the composite-variable factor scores were screened for normality. None deviated significantly as indicated by skewness values<±1. Table 2 presents means and s.d. for the latent variable factor scores, and for age and BMI, reported separately for female and male participants. Group differences were assessed by a one-factor multivariate analysis of variance. Results indicated—in accord with previous research—that females reported a greater tendency to overeat,^69, 70^ and a more pronounced sensitivity to punishment,^71^ whereas males demonstrated a great sensitivity to reward.^71, 72^

### Genotype frequencies and endophenotype analyses

Table 3 lists the genotype frequencies for the seven *OXTR* SNPs used in the following analyses. None of the genotypes for the seven *OXTR* SNPs deviated significantly from Hardy–Weinberg equilibrium (*P*>0.05). Owing to the low frequency of the minor allele for the rs2268493, rs2268494 and rs2254298 SNPs, the homozygous minor-allele group was combined with the heterozygous group to form a binary genotype variable. Additionally, and in light of arguments which favor the reduction of type II errors (that is, the probability of not rejecting the null hypothesis when it is false) in exploratory and preliminary research,^73^ we did not control for multiple testing in the genotype analyses.

There were no group effects for rs53576, rs2254298 and rs237878. However, the remaining four SNPs demonstrated significant genotype differences on at least one endophenotypic variable. Findings for the SNPs with significant effects are summarized in Table 4. For rs2268493, the homozygous T group reported greater overeating than the group with at least one copy of the C allele. With respect to rs2268494, the group with at least one copy of the A allele reported a stronger preference for sweet and fatty foods compared with those with the homozygous T genotype.

For rs2268498 there were main effects for both RS and PS with the homozygous C genotype reporting higher sensitivity on both measures. *Post hoc* comparisons using the least significant difference test indicated that the CC group was higher than both the CT and TT groups (*P*=0.020 and 0.009, respectively) on PS, whereas the CC group was only higher than the CT group regarding the RS variable (*P*=0.015).

There was also a rs227885 main effect for RS indicating higher scores among the G homozygous group. Least significant difference *post hoc* comparisons demonstrated this group had greater scores than both the TT and the TG groups (*P*=0.012 and 0.019, respectively).

### Structural equation modeling

Although the current model did produce a significant *χ*^2^ (32.145, d.f.=14, *P*=0.004), this result is unsurprising given the sample size.^74^ Consequently, it is conventional to examine indices other than the exact-fit *χ*^2^ to determine the quality of the fit of an SEM model. Following is a summary of these statistical indicators.

The relative *χ*^2^ (CMIN/d.f.) measure divides the *χ*^2^ statistic by its degrees of freedom. A value below 3 is deemed an acceptable value for a well-fitting model,^74^ and the current model produced a relative *χ*^2^ of 2.296. The root mean square error of approximation is the most popular measure of goodness-of-fit, with an acceptable fit indicated by values below 0.06 and lower and upper confidence interval limits below 0.05 and 0.08, respectively.^75^ The model had a root mean square error of approximation of 0.051 (0.028, 0.074). The Tucker–Lewis index, comparative fit index, and normed fit index should be at least 0.90, with a well-fitting model scoring 0.95 or greater.^75^ The model resulted in Tucker–Lewis index, comparative fit index, and normed fit index values of 0.870, 0.949 and 0.918, consecutively.

Overall, and according to well-established statistical criteria, the model was therefore a good fit to the data. AMOS produces estimates of *R*^2^ (squared multiple correlations). For overeating, the *R*^2^ value is 0.298 (0.301 when controlling for sex in the model). All parameter estimates were in the expected direction and statistically significant, with the exception of the effect of the rs2268498 SNP on RS (*P*=0.257) and the covariance between the rs2268493 and rs2268498 (*P*=0.966). See Figure 1 for the standardized regression weights. (Note 1: In light of the significant male–female differences in the mean RS, PS and overeating variables, the SEM was rerun adding paths between sex and these three variables. This change made virtually no difference to the fit of the model as seen by the fit indices reported below. Significant pathways resulted from sex to PS (−0.152; *P*=0001), to RS (0.106, *P*=0.028) and to the overeating (−0.087, *P*=0.036) variable. The model fit indices with sex added to the model are: *χ*^2^=2.778, *P*<0.0001, CMIN=49.996, Tucker–Lewis index=0.794, comparative fit index=0.917, normed fit index=0.884, root mean square error of approximation=0.060 (0.040–0.079). As negligible differences resulted in the pathway estimates when controlling for sex, it was decided to leave sex out of the model since the fit indices are somewhat better without sex in the model).

### Haplotype analyses

**Figure 2:**
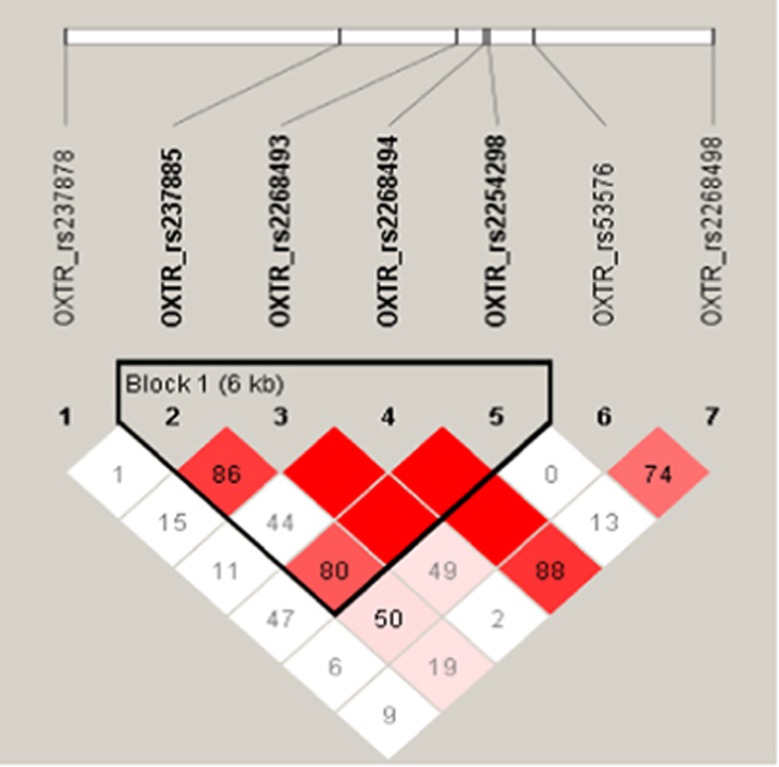
LD plot for the *OXTR* gene SNPs in our sample of Caucasian participants. The values indicate the pairwise LD (*D*′), and the intensity of the color scheme is based on *D*′. The LD block was defined by an *R*^2^ threshold of 0.7 on solid spine.

Linkage disequilibrium analysis showed that the rs237885, rs2268493, rs2268494 and rs2254298 SNPs formed one haplotype block, and rs53576 and rs2268498 formed another, as seen in Figure 2. Analysis of the four-SNP haplotypes indicated that the G–T–A–G haplotype was associated with higher scores on the food preferences composite variable (haplotype window *P*=0.0055; haplotype frequency=6.06% estimated additive value=0.74; 95% confidence interval: 0.32–1.16; haplotype-specific *P*=0.00046). (Note 2: Results in the subsample of Caucasian participants were similar, with the G–T–A–G haplotype being associated with higher scores on the food preference composite score (haplotype window *P*=0.011; haplotype frequency=7.56% estimated additive value=0.64; 95% confidence interval: 0.23–1.06; haplotype-specific *P*=0.00196).

## Discussion

It is well established that large individual differences exist both in basal OXT levels and in the reactivity of the OXT system and the abundance of its receptors—factors that influence responsiveness to social stimuli and that aid in the regulation of food intake.^76^ Generally speaking, a robust OXT system is more resilient and receptive to social reward, and better able to ameliorate anxiety and reduce stress.^76^ These are all characteristics that tend to be diminished in many individuals with chronic overeating.^77^

To the best of our knowledge, this is the first study to examine genetic markers of the OXT system from a broad ‘OXT-deficiency’ perspective in relation to overeating, and endophenotypes of overeating. SEM was used to assess multiple simultaneous relationships in a theoretical model proposing the influence of psychobehavioral traits on overeating, and their underlying biological links as indicated by relevant *OXTR* SNPs. Results indicated that our model was a good fit to the data. All specified paths were statistically significant in the predicted direction except the path between rs2268498 and RS, which did not survive the *P*-value imposed by SEM to account for multiple simultaneous tests. Independent and additive associations, in the positive direction, were found for RS, PS and sugar/fat food preferences on overeating (*R*^2^=30%). To date, only a handful of studies has examined the simultaneous relationships between RS and PS and eating-related problems, and are in accord with the current findings.^78, 79^ Previous evidence also indicates a clear relationship between overeating and elevated BMI, and palatable food preferences.^80, 19^

### OXT associations

The significant association between the G–T–A–G haplotype and high sugar/fat food preferences is supportive of the OXT-deficiency viewpoint in the context of overeating. In previous research, the rs237885 G allele was associated with the highest levels of prepulse inhibition, low depressive symptoms^50^ and lower unemotional traits.^49^ The rs2268493 T allele and the rs2268494 A allele have each been associated with poorer social cognition,^39, 40, 41, 42^ and less emphatic concern.^44^ Similarly, the GG genotype of rs2254298 has also been associated with lower empathic concern and social reciprocity, and lower salivary OXT levels.^44, 81^ Empathy is a key component of prosocial-emotional development with heritability estimates above 50%.^82^ In summary, our haplotype findings suggest that reduced OXT signaling, which is related to social deficits and to lower social responding, may also influence sweet and fatty food preferences. Such a link meshes with the association between low OXT levels and increased sucrose intake.^10^

Single-marker analysis found that four of the seven *OXTR* SNPs included were associated with at least one of the composite-dependent variables. In accord with the haplotype analysis, the A allele carriers at rs2268494 reported higher sweet and fatty food preferences than those with TT. Relatedly, the TT genotype at rs2268493 reported greater overeating compared with the combined CC+CT group. In addition, the GG genotype of the rs237885 marker was related to higher RS compared with the other genotype groups. Finally, the homozygous C group for rs2268498 reported significantly higher RS and PS scores.

It is important to note, in relation to OXT markers, that while there is relative consistency across studies concerning a marker’s association with a particular trait or characteristic, the ‘risk’ allele may differ across samples. That is, the direction of the effect is sometimes inconstant. Such findings support the *social salience* theory of OXT whereby the effects of OXT are nuanced by stable characteristics of the individual and the environmental context,^2^ so that OXT ‘does not cast a rose-colored hue for all’^2^ (p. 105). The role of other individual differences is therefore important when interpreting the role of OXT on psychobehavioral outcomes,^83^ and an important step forward for future researchers in this area of investigation.

In conclusion, this study presents an innovative psychogenetic risk model of overeating and its relevant endophenotypic correlates. As such, it has several strengths including a sound model based on theoretically and empirically supported relationships. Nevertheless, it is important to also address some limitations in this research. Importantly, replication is essential in all SNP–association studies to strengthen confidence in the current findings. As this was the first study to examine relationships between overeating and its endophenotypes, and markers of the *OXTR* gene, we hope that other investigators will attempt to confirm our findings in future research. Relatedly, it is necessary to assess findings from Caucasian-specific single-SNP analysis in larger independent samples. As mentioned in the Note 2 above, however, the direction of the current findings were not changed, and remained significant, when the haplotype analysis was carried out in the Caucasian-only sample.

## Figures and Tables

**Table 1:** Component matrices for the four composite variables

*Latent variable*	*Factor loadings*	*% Explained variance*
*Reward sensitivity (*n=*424)*		57.0
1. IMP	0.65	
2. SPSRQ	0.86	
3. BAS	0.74	
		
*Punishment sensitivity (*n=*423)*		79.4
1. Harm Avoidance (TPQ)	0.90	
2. SPSRQ	0.90	
3. BIS	0.87	
		
*Food-reward preferences (*n=*432)*		87.32
1. High Sugar Foods	0.91	
2. High Fat Foods	0.95	
3. High Sugar and High Fat Foods	0.94	
		
*Overeating (*n=*427)*		70.0
1. Binge eating	0.84	
2. Emotional eating	0.89	
3. Snacking on sweets	0.76	

Abbreviations: BAS, Behavioral Activation Scale; BIS, Behavioral Inhibition Scale; IMP, Barrett Impulsivity Scale; SPSRQ, Sensitivity to Punishment and Sensitivity to Reward Questionnaire.

**Table 2:** Means and s.d. for all quantitative variables included in the study, listed separately for male and female participants

*Variable*	*Females*		*Males*		F	P-*value*
	N	*Means (s.d.)*	N	*Means (s.d.)*		
BMI (kg m^−2^)	346	32.4 (9.5)	114	32.2 (8.8)	0.06	0.799
Age	346	33.4 (6.6)	115	34.3 (7.0)	1.92	0.166
Reward-Sensitivity Factor Score	318	−0.06 (0.97)	106	0.18 (1.1)	4.88	**0.028**
Punishment-Sensitivity Factor Score	317	0.09 (0.98)	106	−0.27 (1.0)	10.68	**0.001**
Food Preference Factor Score	324	−0.04 (1.0)	106	0.13 (0.98)	2.47	0.117
Overeating Factor Score	320	0.06 (0.98)	107	−1.9 (1.0)	5.02	**0.026**

Abbreviation: BMI, body mass index. The bold text indicates and highlights the statistically significant findings.

**Table 3:** Genotypes frequencies for the seven *OXTR* SNPs included in the analyses

*rs53576*	*AA*	*AG*	*GG*	*Total*
Frequency	43	213	201	457
*rs2268493*	*CC*	*CT*	*TT*	
Frequency	26	169	261	456
*rs2268494*	*AA*	*AT*	*TT*	
Frequency	5	59	393	457
*rs2268498*	*CC*	*CT*	*TT*	
Frequency	75	237	144	456
*rs237878*	*CC*	*CT*	*TT*	
Frequency	54	184	189	427
*rs2254298*	*AA*	*AG*	*GG*	
Frequency	9	121	325	455
*rs237885*	*TT*	*TG*	*GG*	
Frequency	108	210	123	441

Abbreviations: *OXTR*, oxytocin receptor; SNP, single-nucleotide polymorphism.

**Table 4:** Means and s.d. are presented for the rs2268493, rs2268494, rs2268494 and rs237885 genotypes, with F and *P*-values for one-way ANOVA comparisons between (and among) groups

*Variable*	*CC+CT*	*TT*	F	P-*value*	*Partial* η^*2*^
	*Mean (s.d.)*	*Mean (s.d.)*			
*rs2268493*					
Reward sensitivity	0.005 (0.94)	−0.002 (1.05)	0.01	0.943	
Punishment sensitivity	−0.18 (1.00)	0.02 (1.00)	0.12	0.728	
Food preferences	−0.03 (0.96)	0.02 (1.03)	0.27	0.604	
Overeating	−0.13 (1.02)	0.09 (0.98)	4.83	**0.028**	0.011

*Variable*	*AA+AT*	*TT*	F	P-*value*	*Partial* η^*2*^
	*Mean (s.d.)*	*Mean (s.d.)*			
*rs2268494*					
Reward sensitivity	0.13 (1.00)	−0.02 (1.00)	1.22	0.270	
Punishment sensitivity	0.10 (0.99)	−0.01 (1.00)	0.62	0.430	
Food preferences	0.29 (0.91)	−0.05 (1.00)	6.11	**0.014**	0.014
Overeating	0.01 (0.93)	−0.001 (1.01)	0.01	0.910	

*Variable*	*CC*	*CT*	*TT*	F	P-*value*	*Partial* η^*2*^
	*Mean (s.d.)*	*Mean (s.d.)*	*Mean (s.d.)*			
*rs2268498*						
Reward sensitivity	0.24 (1.00)	−0.10 (0.98)	0.04 (1.02)	3.14	**0.044**	0.02
Punishment sensitivity	0.29 (1.01)	−0.03 (0.97)	−0.09 (1.03)	3.69	**0.026**	0.02
Food preferences	0.11 (1.10)	0.01 (0.99)	−0.06 (0.98)	0.60	0.548	
Overeating	0.10 (0.95)	−0.05 (0.99)	0.05 (1.03)	0.77	0.462	


*Variable*	*TT*	*TG*	*GG*	F	P-*value*	*Partial* η^*2*^
	*Mean (s.d.)*	*Mean (s.d.)*	*Mean (s.d.)*			
*rs227885*						
Reward sensitivity	−0.12 (1.10)	−0.05 (0.96)	0.23 (0.95)	3.86	**0.022**	0.019
Punishment sensitivity	−0.01 (1.05)	0.05 (1.01)	−0.10 (0.93)	0.83	0.436	
Food preferences	−0.12 (1.12)	0.04 (0.98)	0.05 (0.94)	0.96	0.382	
Overeating	0.10 (0.99)	0.01 (0.98)	−0.10 (1.03)	1.03	0.359	

Abbreviation: ANOVA, analysis of variance.

For each SNP, reward sensitivity (*n*=421), punishment sensitivity (*n*=420), food preferences (*n*=427) and overeating (*n*=422) were the dependent variables. *η*^2^ statistics are only listed for significant effects. The bold text indicates and highlights the statistically significant findings.
